# M*iR-190b*, the highest up-regulated miRNA in ERα-positive compared to ERα-negative breast tumors, a new biomarker in breast cancers?

**DOI:** 10.1186/s12885-015-1505-5

**Published:** 2015-07-05

**Authors:** Geraldine Cizeron-Clairac, François Lallemand, Sophie Vacher, Rosette Lidereau, Ivan Bieche, Celine Callens

**Affiliations:** Service de Génétique, Unité de Pharmacogénomique, Institut Curie, 26 rue d’ulm, 75005 Paris, France

**Keywords:** Breast cancer, MicroRNA, Estrogen receptor, *miR-190b*

## Abstract

**Background:**

MicroRNAs (miRNAs) show differential expression across breast cancer subtypes and have both oncogenic and tumor-suppressive roles. Numerous microarray studies reported different expression patterns of miRNAs in breast cancers and found clinical interest for several miRNAs but often with contradictory results. Aim of this study is to identify miRNAs that are differentially expressed in estrogen receptor positive (ER^+^) and negative (ER^−^) breast primary tumors to better understand the molecular basis for the phenotypic differences between these two sub-types of carcinomas and to find potential clinically relevant miRNAs.

**Methods:**

We used the robust and reproductive tool of quantitative RT-PCR in a large cohort of well-annotated 153 breast cancers with long-term follow-up to identify miRNAs specifically differentially expressed between ER^+^ and ER^−^ breast cancers. Cytotoxicity tests and transfection experiments were then used to examine the role and the regulation mechanisms of selected miRNAs.

**Results:**

We identified a robust collection of 20 miRNAs significantly deregulated in ER^+^ compared to ER^−^ breast cancers : 12 up-regulated and eight down-regulated miRNAs. *MiR-190b* retained our attention as it was the miRNA the most strongly over-expressed in ER^+^ compared to ER^−^ with a fold change upper to 23. It was also significantly up-regulated in ER^+^/Normal breast tissue and down-regulated in ER^−^/Normal breast tissue. Functional experiments showed that *miR-190b* expression is not directly regulated by estradiol and that *miR-190b* does not affect breast cancer cell lines proliferation. Expression level of *miR-190b* impacts metastasis-free and event-free survival independently of ER status.

**Conclusions:**

This study reveals *miR-190b* as the highest up-regulated miRNA in hormone-dependent breast cancers. Due to its specificity and high expression level, *miR-190b* could therefore represent a new biomarker in hormone-dependent breast cancers but its exact role carcinogenesis remains to elucidate.

**Electronic supplementary material:**

The online version of this article (doi:10.1186/s12885-015-1505-5) contains supplementary material, which is available to authorized users.

## Background

Breast cancer is the leading cause of cancer death in women worldwide. Despite advances in the understanding of cancer pathogenesis and improvement in diagnosis and treatment over the past few decades, biomarkers of clinical interest are not so numerous. Now it is well documented that endogenous estrogens known as an important regulator of development, growth and differentiation of the normal mammary gland play also a major role in the development and progression of breast cancer [1]. The mammary cell proliferation signals are mediated in part by the estrogen receptor alpha (ER). The expression of ER in breast tumors is frequently used to separate breast cancer patients in a clinical setting both as an important prognostic marker for prognosis and in predicting the likelihood of response to endocrine therapy. Although the majority of primary breast cancers are ER-positive (ER^+^) and respond well to antiestrogen therapy, up to one-third of patients with breast cancer lack ER (ER^−^) at the time of diagnosis, and a fraction of breast cancers that are initially ER^+^ lose ER expression during tumor progression [2]. These patients fail to respond to antiestrogen therapy and have higher tumor aggressiveness and poor prognosis. Previous studies have shown that ER absence is a result of hypermethylation of CpG islands in the 5’ region of ER coding gene (*ESR1*) in a fraction of breast cancer [2]. However, the molecular mechanism of the rest of the ER^−^ breast cases and the molecule(s) involving ER hypermethylation remain largely unknown. Other mechanisms involved in altering ER expression have been identified, including mutations within the open reading frame of *ESR1* [3] as well as *ESR1* amplification increasing the ER protein expression [4]. Recently, ESR1 ligand-binding domain mutations were described in hormone-resistant breast cancers [5].

Since their first description in *C. Elegans* in 1993, increasing numbers of studies showing frequent deregulation of microRNAs (miRNAs) in human breast cancers and association of some of them with cancer metastasis and poor prognosis suggesting an important role of miRNAs in cancer development and progression [6, 7]. miRNAs are small non-coding RNA gene products able to regulate gene expression at the post-transcriptional level. Thus, today, miRNAs are increasingly seen as important regulators of gene expression in breast cancers, acting either as oncogenes (such as *miR-21*) or tumor suppressors (such as *let-7*), and affecting through different mechanisms many cellular processes that are routinely altered in cancer, such as differentiation, proliferation, apoptosis, metastasis and telomere maintenance [8–11]. MiRNAs are also thought of as biomarkers in cancer diagnosis and prognosis [12]. The diagnostic potential of circulating miRNAs is based mainly on their non-invasive detection in serum and plasma and on their high resistance under difficult environmental conditions, offering them therefore an emerging role in developing new follow-up markers and strategies for cancer treatment [13–15]. Moreover, studies suggested that expression profiles of miRNAs are informative for the classification of human breast cancers [16–18]. Numerous datas are available regarding the miRNA expression in ER^+^ and ER^−^ breast cancer tissues and come mainly from studies using miRNA microarray techniques [16, 19, 20]. Results and conclusions from these old studies are generally not consistent and sometimes even conflicting. More recently, miRNA landscape in breast cancer was deciphering in a large cohort with matching detailed clinical annotation and long-term follow-up but not particularly taking into account ER^+^ and ER^−^ contexts [17]. Taken together, these finding have prompted us to use the robust quantitative RT-PCR technology to identify miRNAs that are differentially expressed in ER^+^ and ER^−^ in breast primary tumors with the aim to better understand the molecular basis for the phenotypic differences between these two sub-types of carcinomas and to find potential clinically relevant miRNAs.

## Methods

### Patients and samples

Breast tumor samples were obtained from 184 postmenopausal women with primary unilateral non metastatic breast adenocarcinoma who underwent biopsies or initial surgery at the Curie Institute/René Huguenin Hospital (Saint-Cloud, France) between 1984 and 2009. Each patient signed a written informed consent form and this study was approved by the Curie Institute/René Huguenin Hospital ethics committee. Immediately after biopsy or surgery, the tumor samples were stored in liquid nitrogen in −80 °C until RNA extraction. All samples analyzed contained more than 70 % of tumor cells. Tumor samples included 106 ER^+^ and 78 ER^−^ tumors. ER status was determined at the protein level by using biochemical methods (Dextran-coated charcoal method until 1988 and enzyme immunoassay thereafter) and was confirmed at mRNA level by RT-PCR. Control samples consisted of twelve specimens of normal breast tissue obtained from women undergoing cosmetic breast surgery or adjacent normal breast tissue from breast cancer patients [21]. Thirty-one of breast tumor samples, comprising 21 ER^+^ and 10 ER^−^, as well as 8 normal breast samples, were used as a RT-PCR pan-miRNA screening set to identify and select miRNAs differentially expressed in ER^+^ compared to ER^−^. These selected miRNAs were then validated in the remaining 153 breast tumor samples comprising 85 ER^+^ and 68 ER^−^ compared to eight normal breast samples. Clinicopathological characteristics of patients in relation to metastatic free survival in the screening and validation series are provided in Table 1. In the screening set, we voluntary included more SBR grade III tumors with the aim to facilitate identification of robust genes differentially expressed whereas the validation set is totally representative of breast cancers treated in the Curie institute/René huguenin hospital between 1984 and 2009.

**Table 1:** Pathological and clinical characteristics of patients in relation to metastasis free survival (MFS) in the screening and validation sets

	Screening set (n = 31)			Validation set (n = 153)		
Characteristic	Number of patients	Number of events (%)	MFS *p*-value^a^	Number of patients	Number of events (%)	MFS *p*-value^a^
Age			0.6228			0.0184
≤65 years	17	3 (18)		67	33 (49)	
>65 years	14	3 (21)		86	27 (31)	
SBR histological grade^b,c^			0.0453			0.0008
I + II	11	0 (0)		96	31 (32)	
III	19	6 (32)		54	27 (50)	
Lymph node status^c^			0.6825			0.6493
Negative	9	2 (22)		33	11 (33)	
Positive	21	3 (14)		112	47 (42)	
Lymph node status			0.3521			0.0005
0	9	2 (22)		33	11 (33)	
[1–3]	18	2 (11)		83	27 (33)	
>3	3	1 (33)		29	20 (69)	
Macroscopic tumor size^c^			0.4955			0.0267
≤25 mm	20	3 (15)		61	18 (30)	
>25 mm	11	3 (27)		83	40 (48)	
Macroscopic tumor size^c^			0.9925			0.1375
≤30 mm	26	5 (19)		92	34 (37)	
>30 mm	5	1 (20)		52	24 (46)	
Estrogen receptor status^c^			0.2867			0.0005
Negative	10	1 (10)		68	34 (50)	
Positive	21	5 (24)		85	26 (31)	
Progesterone receptor status^c^			0.2136			0.0005
Negative	11	1 (9)		68	34 (50)	
Positive	20	5 (25)		85	26 (31)	
*HER2* status^c^			0.8493			0.0595
Negative	22	5 (23)		111	41 (37)	
Positive	5	1 (20)		42	19 (45)	
Treatment^c^			0.6248			0.0393
No treatment	4	0 (0)		13	8 (62)	
Chemotherapy	1	0 (0)		32	14 (44)	
Hormone therapy	21	5 (24)		93	31 (33)	
Chemotherapy and hormone therapy	1	0 (0)		9	6 (67)	

^a^Log-rank test

^b^Scarff Bloom Richardson classification

^c^Histological or treatment information were not available for all tumors

### RNA extraction

Total RNA was extracted from breast tissue by using the acid-phenol guanidium method. Total RNA concentration was quantified using a NanoDrop™ spectrophotometer. RNA quality was determined by agarose gel electrophoresis and ethidium bromide staining. The 18S and 28S RNA bands were visualized under ultraviolet light.

### miRNA expression profiling

MiRNA expression levels in samples were quantified by quantitative RT-PCR (RT-qPCR) using the SYBR Green Master Mix kit on the ABI Prism 7900 Sequence Detection System (Perkin-Elmer Applied Biosystems, Foster City, CA, USA). The Human miScript Primer Assays version 9.0 and 11.0 from Qiagen, designed to detect 804 human miRNA probes, were used according to the manufacturer’s guidelines. Small nucleolar RNA *RNU44* (Qiagen) was used as endogenous control to normalize miRNA expression levels. The relative expression level of each miRNA, expressed as N-fold difference in target miRNA expression relative to*RNU44*, and termed "N*target*", was calculated as follows: N*target* = 2^ΔCt*sample*^. The value of the cycle threshold (ΔCt) of a given sample was determined by subtracting the Ct value of the target miRNA from the average Ct value of *RNU44*. The N*target* values of samples were subsequently normalized such that the median N*target* value of normal breast samples was one. To overcome limits of detection of RT-qPCR, and be sure in expression values of miRNAs, we have considered a miRNA as relevant when the Ct values were lower than 30 in at least 50 % of all samples analyzed. The relative expression of each miRNA was characterized by the median and the range, and a non-parametric Mann–Whitney test was used for statistical analysis of differences in miRNA expression between groups.

### Gene expression profiling

In the validation series, mRNA expression levels of *Dicer* (NM_177438), *Drosha* (NM_013235), *AGO2* (NM_012154), *DGCR8* (NM_022720), four protein-coding genes required to the miRNA biogenesis, and six host genes *CTDSPL*(NM_005808.2),*EVL*(NM_016337.2), *NFYC*(NM_014223.4) *OGFRL1*(NM_024576.3),*CTDSP1* (NM_021198.1), PTMA (NM_002823.4) containing the identified miRNAs were measured by RT-qPCR. Primers and PCR conditions are available on request, and the RT-qPCR protocol is described above. The mRNA expression level of each protein-coding gene is relative to the *TBP* gene (NM_003194).

### Breast cancer cell lines

Expression levels of selected miRNAs were measured by RT-qPCR in a collection of RNAs from 30 human breast cancer cell lines commonly used including 19 ER^−^ (BT-20, BT-549, HCC-38, HCC-70, HCC-202, HCC-1143, HCC-1187, HCC-1569, HCC-1599, HCC-1937, HCC-1954, Hs-578 T, MDA-MB-157, MDA-MB-231, MDA-MB-435 s, MDA-MB-436, MDA-MB-453, MDA-MB-468 and SK-BR-3) and 11 ER^+^ (BT-474, BT-483, CAMA1, HCC-1428, HCC-1500, MCF-7, MDA-MB-134VI, MDA-MB-361, MDA-MB-415, T-47D and ZR-75-1). These RNAs were provided by the transfer department of Curie Institute. For each miRNA and each cell line, mRNA levels were normalized such that the median value of the ER^−^ breast cancer cell lines was one.

The effects of 17β-estradiol (E2) on the miRNA expression were studied on two ERα-positive breast cancer cell lines whose growth is known to be stimulated by E2 : MCF-7 cell line for all selected miRNAs and T-47D cell line for *miR-190b*. They were cultured in either minimum essential medium (MEM) or Dulbecco’s modified Eagle medium (DMEM) supplemented with 10 % fetal calf/bovine serum and antibiotics (penicillin 50 g/ml, streptomycin 50 g/ml and neomycin 100 g/ml) at 37 °C with 5 % CO2. For experiments using E2, MCF-7 and T-47D were grown in phenol red-free minimum essential medium (MEM) supplemented with 5 % charcoal-dextran-stripped fetal calf serum for at least 3 days before treatment. The cells were then treated with E2 (Sigma) diluted in ethanol (EtOH) at 1 nM for MCF-7 and 10 nM for T-47D, or with vehicle EtOH (control cells). RNAs were extracted from these cells after 6 h, 18 h and 4 days of the presence of E2 and the mRNA levels measured by RT-PCR were normalized such that the median value of control cells was of one. Three independent experiments were realized for each time and each condition. To verify the effects of E2 on growth of cells, mRNA expression of pS2/TFF1 (NM_003225), a well-known ERα-induced gene, was also measured by RT-qPCR on the treated cells.

The effects of *miR-190b* expression on cellular proliferation were studied on breast cancer cell lines ER^+^ MCF-7 and T-47D that were transfected with antagomir against *miR-190b* (sequence complementary to *miR-190b* which blocks its effect) and on breast cancer cell line ER^−^ MD-MBA-231 that was transfected with a *miR-190b* mimic (double-stranded RNA which mimics mature endogenous *miR-190b*) using a 3-(4,5-dimethyl-2-thiazolyl)-2,5-diphenyl-2H-tetrazolium bromide (MTT) proliferation assay. In brief, after transient transfection of cells for 24 h with 40 nM of antagomir against *miR-190b* or mimic of *miR-190b* (synthetized by Qiagen), the cells were growth in normal medium for 48, 72 or 120 h to be then treated with 0.5 mg/ml of the MTT labeling reagent at 37 °C for 1 to 3 h and lysed in 150 μl of dimethyl sulfoxide at room temperature for 30 min. The cell viability was thus determined by reading the absorbance at 450 to 570 nm of signal generated by MTT reduction which is directly proportional to the cell number. For each cell line, the data were collected from three independent experiments and compared to the control group obtained by transfection of non-targeting siRNA as negative control in miRNA inhibition experiments or miRNA inhibitor as negative control in miRNA mimic experiment.

### Survival analysis

Metastasis-free survival (MFS) was determined as the interval between initial diagnosis and detection of the first metastasis. Survival distributions were estimated by the Kaplan-Meier method, and the significance of differences between survival rates was ascertained with the log-rank test. The Cox proportional hazards regression model was used to assess prognostic significance, and the results are presented as hazard ratios and 95 % confidence intervals (CIs). Statistical analyses were performed using GraphPad Prism 5 software.

## Results

### Differential miRNA expression between ER^+^ and ER^−^ breast tumors

To identify miRNA expression profiles in breast cancer according to ER status, expression levels of 804 miRNAs were measured by RT-qPCR technology in a well-defined series of 21 ER^+^ and 10 ER^−^ breast tumors and in 8 normal breast tissues (Additional file 1: Table S1). MiRNAs with high Ct values in this screening set and miRNAs with very low expression levels (indicated by an asterisk after their name) were not more studied, resulting in a list of 333 informative miRNAs (Additional file 2: Table S2).

Among these 333 miRNAs a Mann–Whitney test identified 155 miRNAs that were significantly differently expressed in ER^+^ compared to ER^−^ tumors with a *p*-value < 0.05 : 15 miRNAs were up-regulated and 140 miRNAs were down-regulated. We then selected miRNAs that were the most strongly deregulated and for which the specificity of RT-qPCR amplification was verified on the dissociation curve for RT-qPCR validation in a larger independent series of breast tumors. Thus, we focused our study on 11 miRNAs for which the expression level was increased by 2-fold in ER^+^ compared to ER^−^ tumors and 7 miRNAs for which the expression level was decreased by 4-fold in ER^+^ compared to ER^−^tumors (Table 2).

**Table 2:** 18 miRNAs significantly differentially expressed between ER^+^ and ER^−^ breast tumors in the screening series

Official name	Normal breast tissue (n = 8)	ER^+^ breast tumors (n = 21)	ER^−^ breast tumors (n = 10)	ER^+^/ER^−^	
				FC	*p*-value
11 miRNAs up-regulated in ER^+^ compared to ER^−^ with a FC > 2					
*miR-190b*	1.0 (0.06-3.33)	14.5 (2.41-51.7)	0.46 (0.07-6.33)	31.26	<0.0001
*miR-101-1*	1.0 (0.00-2.21)	0.40 (0.05-1.23)	0.08 (0.01-0.48)	4.94	0.0033
*miR-193b*	1.0 (0.12-2.65)	1.87 (0.20-8.68)	0.48 (0.24-2.37)	3.89	0.0106
*miR-342-5p*	1.0 (0.63-1.74)	2.61 (0.30-7.37)	0.94 (0.32-2.87)	2.77	0.0296
*miR-376c*	1.0 (0.00-3.40)	0.53 (0.19-1.40)	0.20 (0.01-0.59)	2.60	0.0083
*miR-451*	1.0 (0.01-3.47)	0.15 (0.04-1.78)	0.06 (0.00-0.19)	2.55	0.0019
*miR-143*	1.0 (0.01-2.24)	0.26 (0.10-1.11)	0.10 (0.01-0.35)	2.52	0.0094
*miR-30c2*	1.0 (0.04-8.70)	1.99 (0.15-11.3)	0.87 (0.07-3.41)	2.29	0.0329
*miR-30e*	1.0 (0.11-9.13)	3.02 (0.29-13.6)	1.41 (0.08-5.73)	2.15	0.0405
*miR-26a1*	1.0 (0.09-2.57)	0.59 (0.28-3.86)	0.28 (0.08-0.86)	2.10	0.0014
*miR-26b*	1.0 (0.08-2.57)	0.52 (0.21-2.79)	0.25 (0.04-0.66)	2.10	0.0050
7 miRNAs down-regulated in ER^+^ compared to ER^−^ with a FC > 4					
*miR-654-3p*	1.0 (0.15-4.36)	0.58 (0.10-4.89)	4.25 (0.68-41.6)	−7.29	0.0008
*miR-203*	1.0 (0.16-3.97)	1.54 (0.12-48.9)	8.86 (1.30-36.4)	−5.76	0.0073
*miR-146a*	1.0 (0.07-2.95)	0.70 (0.07-4.48)	3.71 (0.27-15.3)	−5.30	0.0106
*miR-494*	1.0 (0.24-3.18)	0.20 (0.03-1.68)	0.99 (0.11-1.86)	−4.97	0.0191
*miR-338-5p*	1.0 (0.51-5.60)	0.40 (0.19-1.07)	1.92 (0.67-3.41)	−4.82	<0.0001
*miR-891a*	1.0 (0.24-2.43)	0.34 (0.17-1.28)	1.63 (0.28-2.81)	−4.77	0.0025
*miR-1244*	1.0 (0.22-3.30)	1.12 (0.09-2.79)	4.86 (0.76-17.5)	−4.33	0.0050

Results in ER^+^ and ER^−^ tumors are expressed as the median (range) of miRNA level relative to normal breast tissues and the difference in miRNA expression between ER^+^ and ER^−^ were analysed for significance with the Mann–Whitney test. The miRNAs are ranked according to the fold change (FC) calculated between ER^+^ and ER^−^

### miRNAs associated with ER status in an independent validation series

The expression levels of these 18 miRNAs selected in the screening series were then verified in a validation series including 153 breast tumors (85 ER^+^ and 68 ER^−^) and eight normal breast tissues (Table 3).

**Table 3:** Relative miRNA expression levels of the 30 selected miRNAs between ER^+^ and ER^−^ breast tumors in the validation series

Official name	Normal breast tissue (n = 8)	ER^+^ breast tumors (n = 85)	ER^−^breast tumors (n = 68)	ER^+^/ER^−^	
				FC	*p*-value
11 up-regulated miRNAs selected in the screening series					
*miR-190b*	1.0 (0.32-1.57)	6.34 (0.71-32.3)	0.27 (0.02-6.23)	23.30	<0.0001
*miR-101-1*	1.0 (0.69-8.05)	0.30 (0.05-2.10)	0.15 (0.01-0.92)	2.01	<0.0001
*miR-193b*	1.0 (0.43-1.79)	1.44 (0.10-9.17)	1.00 (0.11-4.45)	1.43	0.0191
*miR-342-5p*	1.0 (0.78-2.16)	2.03 (0.29-19.4)	0.93 (0.10-3.65)	2.18	<0.0001
*miR-376c*	1.0 (0.52-3.04)	0.14 (0.02-1.55)	0.10 (0.02-0.83)	1.43	0.0064
*miR-451*	1.0 (0.32-11.1)	0.17 (0.02-14.0)	0.14 (0.01-9.77)	1.23	ns
*miR-143*	1.0 (0.45-2.72)	0.21 (0.03-1.40)	0.14 (0.02-0.74)	1.52	0.0140
*miR-30c2*	1.0 (0.69-1.72)	0.54 (0.12-3.06)	0.34 (0.09-1.17)	1.60	<0.0001
*miR-30e*	1.0 (0.70-2.98)	0.79 (0.12-4.57)	0.47 (0.10-2.28)	1.68	<0.0001
*miR-26a1*	1.0 (0.77-4.06)	0.34 (0.11-1.78)	0.09 (0.02-0.47)	3.64	<0.0001
*miR-26b*	1.0 (0.68-4.38)	0.49 (0.15-2.93)	0.34 (0.06-1.64)	1.44	0.0008
7 down-regulated miRNAs selected in the screening series					
*miR-654-3p*	1.0 (0.72-1.34)	0.30 (0.05-2.68)	0.66 (0.04-17.8)	−2.16	<0.0001
*miR-203*	1.0 (0.51-2.43)	0.88 (0.02-5.78)	1.51 (0.05-19.6)	−1.72	0.0059
*miR-146a*	1.0 (0.54-3.71)	0.70 (0.07-3.63)	0.95 (0.10-4.30)	−1.36	0.0344
*miR-494*	1.0 (0.85-2.17)	0.65 (0.02-6.96)	0.69 (0.02-9.03)	−1.07	ns
*miR-338-5p*	1.0 (0.79-1.78)	0.84 (0.19-4.66)	0.96 (0.12-5.08)	−1.15	ns
*miR-891a*	1.0 (0.77-1.64)	1.37 (0.14-8.43)	2.07 (0.37-15.5)	−1.51	ns
*miR-1244*	1.0 (0.73-1.69)	1.23 (0.16-5.11)	2.34 (0.35-25.3)	−1.91	<0.0001
12 miRNAs selected from the literature					
*let-7a*	1.0 (0.73-1.79)	0.58 (0.15-2.00)	0.47 (0.11-2.99)	1.24	0.0055
*let-7b*	1.0 (0.71-1.69)	0.53 (0.08-2.04)	0.31 (0.04-0.82)	1.75	<0.0001
*miR-18a*	1.0 (0.49-3.33)	0.50 (0.06-2.53)	1.12 (0.12-23.9)	−2.24	<0.0001
*miR-18b*	1.0 (0.47-4.45)	0.50 (0.07-3.52)	1.04 (0.13-25.3)	−2.07	<0.0001
*miR-19a*	1.0 (0.70-2.13)	0.34 (0.03-2.61)	0.42 (0.02-14.4)	−1.24	ns
*miR-21*	1.0 (0.49-5.26)	2.05 (0.41-16.6)	1.84 (0.21-9.53)	1.12	ns
*miR-22*	1.0 (0.38-4.51)	0.71 (0.12-12.9)	0.66 (0.09-3.36)	1.08	ns
*miR-92a1*	1.0 (0.68-1.40)	0.32 (0.10-1.17)	0.49 (0.09-8.95)	−1.54	<0.0001
*miR-155*	1.0 (0.55-4.24)	2.06 (0.52-10.9)	3.97 (0.35-32.0)	−1.93	<0.0001
*miR-206*	1.0 (0.01-2.28)	0.25 (0.02-7.71)	0.32 (0.01-2.74)	−1.29	ns
*miR-221*	1.0 (0.65-1.92)	0.40 (0.07-4.24)	0.53 (0.05-5.55)	−1.31	ns
*miR-222*	1.0 (0.63-2.30)	0.39 (0.06-2.68)	0.50 (0.04-3.88)	−1.28	ns

Results in ER^+^ and ER^−^ tumors are expressed as the median (range) of miRNA level relative to normal breast tissues. For each miRNA, we report the fold-change (FC) between ER^+^ and ER^−^ tumors and the *p*-value associated to Mann–Whitney test (ns for not significant)

In these validation series, we also measured the expression levels of 12 miRNAs reported by the literature to be particularly deregulated in ER^+^ breast tumors : *let-7a* and *let-7b* [22, 23], *miR-18a* and *miR-18b* [24], *miR-21* [25], *miR-22* [26], *miR-155* [27, 28], *miR-206* [29] *and mir-221 and 222* [30] as well as *miR-19a* and *miR-92a1*, which, with *miR-18a*, belonged to the miR-17-92 cluster [31] (Table 3).

Among the 11 up-regulated miRNAs selected from the screening series, except *miR-451*, we validated the up-regulation of *miR-190b*, *miR-101-1, miR-193b*, *miR-342-5p*, *miR-376c, miR-143*, *miR-30c2*, *miR-30e*, *miR-26a1* and *miR-26b* in ER^+^ compared to ER^−^ (Table 3). Among the 12 miRNAs selected from the literature, we found 2 other miRNAs up-regulated in ER^+^ compared to ER^−^: *let-7a1* and *let-7b*. However among these 12 up-regulated miRNAs, we identified 5 different expression profiles according to their expression in ER^+^/Normal and ER^−^/Normal. Eight miRNAs (*miR-26a1*, *miR-101-1*, *let-7b*, *miR-30c2*, *miR-143*, *miR-26b*, *miR-376c* and *let-7a1*) showed a significant decrease of their expression in both ER^+^ and in ER^−^ compared to normal breast tissue (see miR-26a1 for example in Additional file 3: Figure S1A) but with a significantly greater decrease in ER^−^/Normal than in ER^+^/Normal (FC ranging from 2.1 to 11.1 and from 1.7 to 7.1, respectively) (Table 4). The down-regulation of *miR-30e* was specific to ER^−^ (Additional file 3: Figure S1B) since its expression was not differentially expressed in ER^+^/Normal but significantly under-expressed in ER^−^/Normal. *MiR-193b* did not particularly retain our attention to the extent that this miRNA was deregulated neither in ER^+^/Normal nor in ER^−^/Normal (Additional file 3: Figure S1C). *MiR-342-5p* was significantly up-regulated in ER^+^/Normal but not differentially expressed in ER^−^/Normal (Additional file 3: Figure S1D), revealing a specific up-regulation of *miR-342-5p* in ER^+^. Finally, *miR-190b* retained our attention as it was the miRNA the most strongly over-expressed in ER^+^ compared to ER^−^ with a FC upper to 23, much higher than FC of other up-regulated miRNAs (Table 3, Additional file 3: Figure S1E). Moreover the ER^+^ breast tumors showed a *miR-190b* up-regulation compared to normal breast tissue with a FC of 6.34 whereas the ER^−^ breast tumors, a down-regulation with a FC of −3.70 (Table 4).

**Table 4:** Relative miRNA expression levels of the 30 selected miRNAs between ER^+^ or ER^−^ breast tumors and normal breast tissues

Official Name	Normal breast tissue (n = 8)	ER^+^ breast tumors (n= 85)	ER^+^/Normal		ER^−^ breast tumors (n= 68)	ER^−^/Normal	
			FC	*p*-value		FC	*p*-value
12 miRNAs up-regulated in ER^+^ compared to ER^−^							
*miR-190b*	1.0 (0.32-1.57)	6.34 (0.71-32.3)	6.34	<0.0001	0.27 (0.02-6.23)	−3.70	0.0284
*miR-26a1*	1.0 (0.77-4.06)	0.34 (0.11-1.78)	−2.94	<0.0001	0.09 (0.02-0.47)	−11.11	<0.0001
*miR-342-5p*	1.0 (0.78-2.16)	2.03 (0.29-19.4)	2.03	0.0026	0.93 (0.10-3.65)	−1.07	ns
*miR-101-1*	1.0 (0.69-8.05)	0.30 (0.05-2.10)	−3.33	<0.0001	0.15 (0.01-0.92)	−6.67	<0.0001
*let-7b*	1.0 (0.71-1.69)	0.53 (0.08-2.04)	−1.89	0.0007	0.31 (0.04-0.82)	−3.23	<0.0001
*miR-30e*	1.0 (0.70-2.98)	0.79 (0.12-4.57)	−1.27	ns	0.47 (0.10-2.28)	−2.13	0.0005
*miR-30c2*	1.0 (0.69-1.72)	0.54 (0.12-3.06)	−1.85	0.0032	0.34 (0.09-1.17)	−2.94	<0.0001
*miR-143*	1.0 (0.45-2.72)	0.21 (0.03-1.40)	−4.76	<0.0001	0.14 (0.02-0.74)	−7.14	<0.0001
*miR-26b*	1.0 (0.68-4.38)	0.49 (0.15-2.93)	−2.04	0.0034	0.34 (0.06-1.64)	−2.94	0.0001
*miR-376c*	1.0 (0.52-3.04)	0.14 (0.02-1.55)	−7.14	<0.0001	0.10 (0.02-0.83)	−10.00	<0.0001
*miR-193b*	1.0 (0.43-1.79)	1.44 (0.10-9,17)	1.44	ns	1.00 (0.11-4.45)	1.00	ns
*let-7a*	1.0 (0.73-1.79)	0.58 (0.15-2.00)	−1.72	0.0017	0.47 (0.11-2.99)	−2.13	0.0002
8 miRNAs down-regulated in ER^+^ compared to ER^−^							
*miR-18a*	1.0 (0.49-3.33)	0.50 (0.06-2.53)	−2.00	0.0074	1.12 (0.12-23.9)	1.12	ns
*miR-654-3p*	1.0 (0.72-1.34)	0.30 (0.05-2.68)	−3.33	0.0001	0.66 (0.04-17.8)	−1.51	ns
*miR-18b*	1.0 (0.47-4.45)	0.50 (0.07-3.52)	−2.00	0.0063	1.04 (0.13-25.3)	1.04	ns
*miR-155*	1.0 (0.55-4.24)	2.06 (0.52-10.9)	2.06	0.0181	3.97 (0.35-32.0)	3.97	0.0019
*miR-1244*	1.0 (0.73-1.69)	1.23 (0.16-5.11)	1.23	ns	2.34 (0.35-25.3)	2.34	0.0073
*miR-203*	1.0 (0.51-2.43)	0.88 (0.02-5.78)	−1.14	ns	1.51 (0.05-19.6)	1.51	ns
*miR-92a1*	1.0 (0.68-1.40)	0.32 (0.10-1.17)	−3.12	<0.0001	0.49 (0.09-8.95)	−2.04	0.0007
*miR-146a*	1.0 (0.54-3.71)	0.70 (0.07-3.63)	−1.43	ns	0.95 (0.10-4.25)	−1.05	ns
10 miRNAs not differentially expressed in ER^+^ compared to ER^−^							
*miR-451*	1.0 (0.32-11.1)	0.17 (0.02-14.0)	−5.88	0.0009	0.14 (0.01-9.77)	−7.14	0.0003
*miR-21*	1.0 (0.49-5.26)	2.05 (0.41-16.6)	2.05	0.0269	1.84 (0.21-9.53)	1.84	ns
*miR-22*	1.0 (0.38-4.51)	0.71 (0.12-12.9)	−1.41	ns	0.66 (0.09-3.36)	−1.51	ns
*miR-494*	1.0 (0.85-2.17)	0.65 (0.02-6.96)	−1.54	0.0462	0.69 (0.02-9.03)	−1.45	0.0449
*miR-338-5p*	1.0 (0.79-1.78)	0.84 (0.19-4.66)	−1.19	ns	0.96 (0.12-5.08)	−1.04	ns
*miR-19a*	1.0 (0.70-2.13)	0.34 (0.03-2.61)	−2.94	0.0003	0.42 (0.02-14.4)	−2.38	0.0037
*miR-222*	1.0 (0.63-2.30)	0.39 (0.06-2.68)	−2.56	0.0001	0.50 (0.04-3.88)	−2.00	0.0015
*miR-206*	1.0 (0.01-2.28)	0.25 (0.02-7.71)	−4.00	0.0156	0.32 (0.01-2.74)	−3.12	0.0114
*miR-221*	1.0 (0.65-1.92)	0.40 (0.07-4.24)	−2.50	0.0004	0.53 (0.05-5.55)	−1.89	0.0098
*miR-891a*	1.0 (0.77-1.64)	1.37 (0.14-8.43)	1.37	ns	2.07 (0.37-15.5)	2.07	0.0381

Results in ER^+^ and ER^−^ breast tumors are expressed as the median (range) of miRNA level relative to normal breast tissues. For each miRNA, we report the fold-change (FC) between ER^+^ or ER^−^ breast tumors and normal breast tissue and the *p*-value associated to Mann-Whitney’s test (ns for not significant)

Among the seven down-regulated miRNAs selected from the screening series, under-expression of four miRNAs, *miR-654-3p*, *miR-203*, *miR-146a* and *miR-1244*, in ER^+^ compared to ER^−^ was confirmed in the validation series (Table 3). Among the miRNAs selected from the literature, we found four other miRNAs down-regulated in ER^+^ compared to ER^−^: *miR-18a*, *miR-18b*, *miR-92a1* and *miR-155*. Among these eight down-regulated miRNAs, we identified five different expression profiles according to their expression in ER^+^/Normal and RE^−^/Normal (Additional file 4 and Table 4). The first profile concerned *miR-18a*, *miR-18b* and *miR-654-3p* (see miR-654-3p for example in Additional file 4: Figure S2A) that were not differentially expressed in ER^−^/Normal but significantly under-expressed in ER^+^/Normal, revealing a specific down-regulation of *miR-18a*, *miR-18b* and *miR-654-3p* in ER^+^ (Table 4). We found two miRNAs that were not differentially expressed in breast cancer, *miR-203* and *miR-146a* (see miR-146a for example in Additional file 4: Figure S2B) and one miRNA, miR-92a1, that was significantly down-regulated in breast cancer (Additional file 4: Figure S2C). The two last expression profiles concerned *miR-155* (Additional file 4: Figure S2D) and *miR-1244* (Additional file 4: Figure S2E). Although these two miRNAs were significantly up-regulated in ER^*−*^ breast cancer compared to normal breast tissue, *miR-155* showed also significant increase of its expression in ER^+^/Normal whereas miR-1244 was not differentially expressed in ER^+^/Normal, revealing a specific up-regulation of *miR-1244* in ER^−^ (Table 4).

### Expression of genes required for miRNAs biogenesis in ER^+^ and ER^−^ breast tumors

The majority of these 20 miRNAs deregulated in ER^+^ breast tumors, except *miR-155* and *miR-1244*, were significantly down-regulated in breast cancers compared to normal breast tissue (Additional file 5: Table S3) so we explored if genes required for miRNAs biogenesis could be deregulated. In the validation series, we measured by RT-qPCR the expression levels of *DICER1*, *DROSHA*, *AGO2* and *DGCR8*, four genes encoding proteins playing pivotal roles in the processing of mature miRNAs. We found that these genes were deregulated in ER^+^ compared to ER^−^ breast tumors: *DICER1*, *DROSHA* and *DGCR8* were significantly under-expressed in ER^−^ while *AGO2* was moderate over-expressed in ER^−^. We did not however observe significant expression changes in ER^+^/Normal for these four genes. On the other hand, we observed a significant under-expression of *DICER1* in ER^−^/Normal (Table 5). These results revealed a deregulation of genes required for miRNA biogenesis in the absence of ER.

**Table 5:** Expression levels of 4 genes required for miRNA biogenesis in breast tissues

	Breast tissue			ER^+^/ER^−^		ER^+^/Normal		ER^−^/Normal	
Gene	Normal (n = 8)	ER^+^ (n = 85)	ER^−^ (n = 68)	FC	*p*-value	FC	*p*-value	FC	*p*-value
*DICER1*	1.0	0.92	0.50	1.85	<0.0001	−1.09	ns	−2.00	0.0004
	(0.79-1.36)	(0.05-3.18)	(0.10-1.94)						
*DROSHA*	1.0	1.27	0.77	1.65	<0.0001	1.27	ns	−1.30	0.0486
	(0.77-1.56)	(0.08-4.91)	(0.09-6.37)						
*DGCR8*	1.0	1.05	0.72	1.46	<0.0001	1.05	ns	−1.39	0.0506
	(0.78-1.56)	(0.19-4.77)	(0.13-3.44)						
*AGO2*	1.0	0.80	1.10	−1.37	0.0433	−1.25	ns	1.10	ns
	(0.56-1.10)	(0.17-3.74)	(0.08-4.74)						

Results in ER^+^ and ER^−^ breast tumors are expressed as the median (range) of level relative to normal breast tissues. For each comparison, we report the fold-change (FC) and the *p*-value associated to Mann-Whitney’s test (ns for not significant)

### Expression of host genes of miRNAs in ER^+^ and ER^−^ breast tumors

Among the 20 miRNAs identified as deregulated in ER^+^ compared to ER^−^ breast tumors, 6 miRNAs are located in intragenic regions: *miR-26a1* in *CTDSPL*, *miR-342-5p* in *EVL*, *miR-30e* in *NFYC*, *miR-30c2* in *OGFRL1*, *miR-26b* in *CTDSP1* and *miR-1244* in *PTMA*. The expression levels of these 6 host genes were then measured, by RT-qPCR, in the validation series (Table 6). These genes showed significant expression difference between ER^+^ and ER^−^similar to their miRNA (Table 4). Thus *CTDSPL*, *EVL*, *NFYC*, *OGFRL1* and *CTDSP1* are more expressed in ER^+^ than in ER^−^ like *miR-26a1*, *miR-342-5p*, *miR-30a*, *miR-30c2* and *miR-26b* respectively, and *PTMAP2* is less expressed in ER^+^ than in ER^−^ like *miR-1244*. Moreover Spearman’s rank correlation analysis revealed a significant and positive correlation between expression of all host genes and its resident miRNA in breast tumors : *miR-26a1* and *CDTSPL* (r = 0.3157, *p* < 0.0001), *miR-342-5p* and *EVL* (r = 0.5931, *p* < 0.0001), *miR-30e* and *NFYC* (r = 0.3157, *p* < 0.0001), *miR-30c2* and *OGFRL1* (r = .02803, *p* = 0.0004), *miR-26b* and *CDTSP1* (r = 0.2502, *p* = 0.0018) and *miR-1244* and *PTMA* (r = 0.2258, *p* = 0.005), indicating a miRNA-host co-transcription. According to ER status, a significant correlation was observed for *miR-342-5p*/*EVL* in ER^+^ (r = 0.3817, *p* = 0.0004) and for *miR-26b*/*CTDSP1* and *miR-1244*/*PTMAP2* in ER^−^ (r = 0.3584, *p* = 0.0029 and r = 0.2822, *p* = 0.0197, respectively) (data not shown).

**Table 6:** Expression levels of host genes containing miRNAs deregulated in ER^+^ compared to ER^−^ breast tumors in the validation series

		Breast tissue			ER^+^/ER^−^	
miRNA	Host gene	Normal (n = 8)	ER^+^ (n = 85)	ER^−^ (n = 68)	FC	*p*-value
*miR-26a1*	*CTDSPL*	1.0	0.95	0.42	2.26	<0.0001
		(0.49-2.20)	(0.21-19.6)	(0.03-7.64)		
*miR-342-5p*	*EVL*	1.0	3.36	0.54	6.17	<0.0001
		(0.89-1.42)	(0.16-26.9)	(0.08-2.77)		
*miR-30e*	*NFYC*	1.0	1.00	0.90	1.11	0.0143
		(0.85-1.42)	(0.06-2.91)	(0.45-2.46)		
*miR-30c2*	*OGFRL1*	1.0	0.67	0.44	1.51	0.0002
		(0.69-1.29)	(0.03-2.20)	(0.08-2.04)		
*miR-26b*	*CTDSP1*	1.0	0.93	0.67	1.39	<0.0001
		(0.66-1.56)	(0.20-3.85)	(0.25-3.11)		
*miR-1244*	*PTMAP2*	1.0	1.41	1.89	−1.34	0.0002
		(0.53-2.04)	(0.00-6.10)	(0.85-12.4)		

Results in ER^+^ and ER^−^ breast tumors are expressed as the median (range) of level relative to normal breast tissues. For each comparison, we report the fold-change (FC) and the *p*-value associated to Mann-Whitney’s test (ns for not significant)

### *miRNA* expression in human breast cancer cell lines

**Fig. 1:**
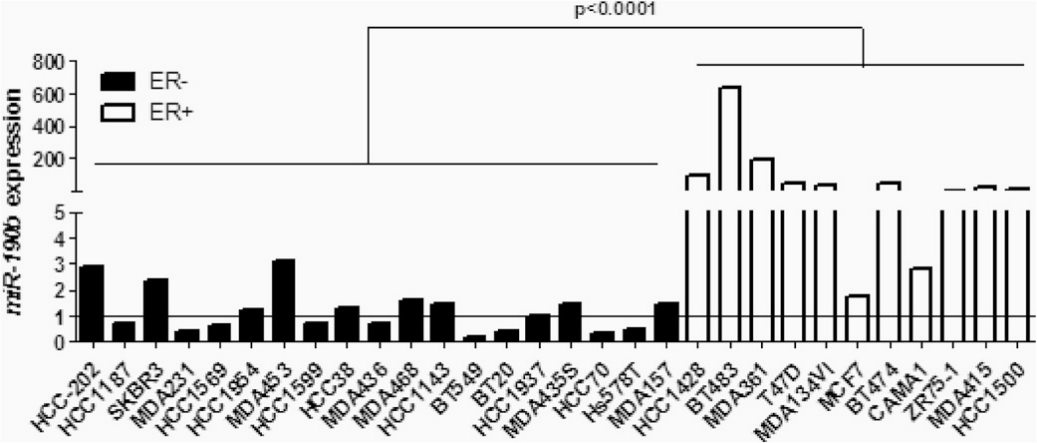
Expression levels of *miR-190b* in breast cancer cell lines. Representation of *miR-190b* relative expression level in 30 breast cancer cell lines. For each cell line, the miRNA levels were normalized such that the median value of the ER^−^ breast cancer cell lines was 1 (horizontal line)

We further evaluated the expression levels of 20 miRNAs identified as deregulated in ER^+^ compared to ER^−^ breast tumors in 30 human breast cancer cell lines including 19 ER^−^ and 11 ER^+^. The patterns of expression changes observed between ER^+^ and ER^−^ breast tumors do not have been validated in breast cancer cell lines for all miRNAs (Table 7). We only confirmed the significant over-expression of *miR-190b*, *miR-26a1*, *miR342-5p*, *miR-101-1*, *miR-26b* and *miR-193b* in ER^+^ breast cancer cell lines. None down-regulation of miRNAs in ER+ compared to ER- tumors was validated in cell lines; *miR-203* was even significantly up-regulated (*p* = 0.0226). It is worthy to note that *miR-190b,* the highest up-regulated miRNA in ER^+^ tumors, was also the highest miRNA expressed in ER^+^ breast cancer cell lines, with a FC of 43 compared to 8 for the second higher up-regulated miRNA, *miR-342-5p* (Table 7), and that this up-regulation was observed in most of ER^+^ breast cancer cell lines (Fig. 1) confirming thus that *miR-190b* may have an important role in ER-dependent tumorigenesis. This is why we decided to focus next experiments on the expression and function of *miR-190b*.

**Table 7:** Relative miRNA expression levels of 20 miRNAs in breast cancer cell lines. Results in breast cancer cell lines are expressed as the median (range) of miRNA level relative to ER- breast cancer cell lines

Official Name	ER^−^ breast cancer cell lines (n = 19)	ER+ breast cancer cell lines (n = 11)	FC	*p*-value
12 miRNAs up-regulated in ER^+^ compared to ER^−^ breast tumors				
*miR-190b*	1.0 (0.18-3.14)	42.9 (1.73-631)	42.99	<0.0001
*miR-26a1*	1.0 (0.36-3.22)	3.14 (0.36-7.83)	3.14	0.0477
*miR-342-5p*	1.0 (0.26-2.72)	8.24 (1.72-17.4)	8.24	<0.0001
*miR-101-1*	1.0 (0.50-2.54)	2.04 (0.61-6.64)	2.04	0.0111
*let-7b*	1.0 (0.34-4.92)	1.02 (0.01-5.24)	1.02	ns
*miR-30e*	1.0 (0.56-3.45)	1.50 (0.68-2.50)	1.50	ns
*miR-30c2*	1.0 (0.43-2.89)	0.92 (0.36-3.94)	−1.09	ns
*miR-143*	1.0 (0.37-36.3)	1.22 (0.37-3.84)	1.22	ns
*miR-26b*	1.0 (0.41-4.89)	3.54 (0.98-7.81)	3.54	0.0006
*miR-376c*	1.0 (0.11-22.8)	0.92 (0.00-22.1)	−1.09	ns
*miR-193b*	1.0 (0.26-3.90)	3.45 (0.29-12.0)	3.45	0.0052
*let-7a*	1.0 (0.50-2.01)	0.87 (0.10-3.61)	−1.15	ns
8 miRNAs down-regulated in ER^+^ compared to ER^−^ breast tumors				
*miR-18a*	1.0 (0.15-2.39)	0.50 (0.24-25.1)	−2.00	0.0642
*miR-654-3p*	1.0 (0.13-9.85)	0.46 (0.24-1.93)	−2.17	ns
*miR-18b*	1.0 (0.17-2.45)	0.64 (0.16-27.5)	−1.56	ns
*miR-155*	1.0 (0.00-72.2)	0.03 (0.00-0.84)	−33.3	0.0609
*miR-1244*	1.0 (0.32-1.90)	0.91 (0.43-2.20)	−1.10	ns
*miR-203*	1.0 (0.01-10.0)	5.62 (0.03-13.3)	5.62	0.0226
*miR-92a1*	1.0 (0.41-2.46)	0.62 (0.35-4.29)	−1.61	0.0707
*miR-146a*	1.0 (0.03-91.6)	0.11 (0.03-0.25)	−9.09	0.0583

The miRNAs are ranked according to the deregulation level between ER^+^ and ER^−^ breast tumors. For each miRNA, we report the fold-change (FC) between ER^+^ and ER^−^ breast cancer cell lines and the *p*-value associated to Mann–Whitney *U* test (ns for not significant)

### Confirmation of *miR-190b* up-regulation in ER^+^ compared to ER^−^ breast tumors

The heightened increase of *miR-190b* expression in ER^+^ compared to ER^−^ breast tumors identified previously by Qiagen quantitative RT-PCR was validated by another experimental technique provided by Applied System Biotechnologies. Indeed, on 20 breast tumor samples which showed a FC of 23 between ER^+^ and ER^−^ with Qiagen technology, we found a similar increase of *miR-190b* expression levels with Applied technology (FC of 26) (data not shown) and a strong positive correlation of *miR-190b* expression between the two techniques (Spearman’s coefficient correlation of 0.8977 significant at *p* < 0.0001).

### Prognostic value of miR-190b expression in breast cancer patients

**Fig. 2:**
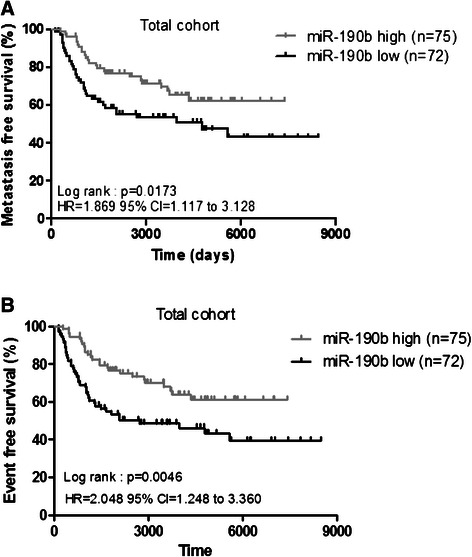
Metastasis-free survival (**a**) and event-free survival (**b**) according to miR-190b expression level in breast tumors for the total cohort. Kaplan-Meier survival analysis stratified by the miR-190b expression level. The p value was determined using the log rank test

Using a Kaplan-Meier analysis, we showed that high expression of *miR-190b* did not impact metastasis-free survival in ER^+^ and ER- separated subgroups (datas not shown). If we compared MFS according to the type of treatment, we observed no prognostic impact related on miR-190b expression level for patients who received hormone therapy alone (*p* = 0.40, datas not shown). All patients receiving other adjuvant treatment expressed miR-190b at low level. Interestingly high expression of miR-190b was associated with a prolonged metastasis-free survival independently to ER status and treatment (log rank test: *p* = 0.0173, HR = 1.869, 95 % CI = 1.12 to 3.13) (Fig. 2A), as well as a prolonged event-free survival (log rank test: *p* = 0.0046, HR = 2.048, 95 % CI = 1.248 to 3.360) (Fig. 2B). This result prompted us to explore functions of *miR-190b* in breast carcinogenesis.

### Effect of estrogen on *miR-190b* expression

To identify if estrogen could explain the deregulation of *miR-190b* between ER^+^ and ER^−^ breast tumors, we measured its expression levels on the ERα-positive MCF-7 and T-47D breast cancer cell lines treated with 17β-estradiol (E2). We did not observe an increase of *miR-190b* expression levels in MCF-7 or in T-47D treated by E2 whereas the expression of the well-known ERα-induced gene *pS2* was highly increased in the two cell lines (Additional files 6: Figure S3A and S3B). Others 19 miRNAs did not respond to 17β-estradiol either (datas not shown). Obviously we neither observed effect of tamoxifen treatment on miR-190b expression in MCF-7 cell lines (datas not shown).

### Role of *miR-190b* expression in tumor proliferation

The heightened increase of *miR-190b* in ER^+^ breast cancer prompted us to explore this possible biological significance in cell proliferation. As initial step, the capacity of proliferation induction was evaluated on breast cancer cell lines ER^+^ MCF-7 and T-47D that were transfected with an antagomir against *miR-190b* and on ER^−^ MD-MBA-231 that was transfected with a *miR-190b* mimic. The efficacy of transfection was verified by quantifying *miR-190b* in RNA extracted from transfected cells by qRT-PCR (datas not shown). Antagomir did not affect proliferation of MCF-7 (Additional file 7: Figure S4A) and T-47D cell lines (Additional file 7: Figure S4B) as *miR-190b* mimic has no effect on MDA-MB-231 proliferation (Additional file 7: Figure S4C). Other experiments are therefore needed to decipher the role of *miR-190b* in mammary tumorigenesis.

## Discussion

From the study of more than 800 miRNA in ER^+^ en ER^−^ breast tumors and confirmation of our results in a large validation series, we identified a robust collection of 20 miRNAs significantly deregulated in ER^+^ compared to ER^−^ breast cancers: 12 up-regulated and eight down-regulated miRNAs. Among these 20 miRNAs, we found ten miRNAs similarly deregulated in ER^+^ and ER^−^, independently to their ER status: *let-7a*, l*et-7b*, *miR-26a1*, *miR-101-1*, *miR-30c2*, *miR-143*, *miR-26b*, *miR-376c* and *miR-92a1* which were down-regulated in both ER^+^/Normal and in ER^−^/Normal and *miR-155* which was up-regulated in both ER^+^/Normal and in ER^−^/Normal. Moreover we found six miRNAs only deregulated in ER^+^/Normal or in ER^−^/Normal (*miR-30e*, specifically down-regulated in ER^−^, *miR-342-5p*, specifically up-regulated in ER^+^, *miR-18a*, *miR-18b* and *miR-654-3p*, specifically down-regulated in ER^+^ and *miR-1244*, specifically up-regulated in ER^−^) and 3 miRNAs, not particularly attractive since not deregulated in ER^+^/Normal nor in ER^−^/Normal (*miR-193b*, *miR-203* and *miR-146a*). In contrast we found a very interesting miRNA, *miR-190b*, which was not only the strongly up-regulated in ER^+^ compared to ER^−^ (FC of 23.30) but also the only miRNA for which deregulation was different in breast cancer according to ER status.

Production and maturation of miRNAs require a set of proteins collectively known as the miRNA biogenesis machinery and it is now established that alterations in this machinery can generate changes in miRNA expression and contribute thus in the development and progression of cancer. We fully support the same view since we found that several key miRNA processing genes are differentially expressed between ER^+^ and ER^−^ breast cancer, which may explain the different regulatory effects of miRNAs in these two breast cancer subtypes. Indeed *DROSHA*, *DGCR8* and *DICER1* were significantly down-regulated in ER^−^ whereas *AGO2* was moderate up-regulated. These results support previous observations [16, 20, 32–34] and strengthen the pertinence of alterations of the basic miRNA biogenesis machinery in breast cancer.

For all six miRNAs located in intragenic regions, we demonstrated a significant and positive correlation between expression of host gene and its resident miRNA, in particular the well-known co-regulation of the ER+ marker miR-342 and the ER-regulated gene *EVL* [16, 35, 36]. However, in the study of Dvinge, results suggested a limited miRNA-host co-transcription concerning only 49 out 227 same-strand intragenic miRNAs [17].

These 20 miRNAs have been already described in early studies identifying miRNAs implicated in breast cancers [7, 9, 15, 17] but *miR-190b* retained our attention because it was the only miRNA strongly up-regulated in ER^+^ compared to ER^−^ breast tumors with a FC of 23 much higher than all other up-regulated miRNAs. This observation remained true also in breast cancer cell lines with a FC of 43 for *miR-190b.* Moreover *miR-190b* was strongly up-regulated in ER^+^ breast tumors compared to normal breast cancer and especially the only miRNA for which deregulation was different according to ER status. Indeed *MiR-190b* was significantly up-regulated in ER^+^/Normal and down-regulated in ER^−^/Normal suggesting a different deregulation of *miR-190b* depending on the ER status. We could note that we did not select *miR-135b* because of absence of expression in our screening series whereas this microRNA would be differentially expressed in ER^+^ and ER^−^ breast tumors, and is described by Aakula *et al*. as a regulator of ER [37]

Few studies reported miR-190b implication in cancers. A recent next generation sequencing project identified mir-190b among seven others microRNAs as a biomarker for the diagnosis of Merkel cell carcinoma [38]. In lung cancer, miR-190b could be detected easily in serum of patients to facilitate diagnosis [39]. MicroRNA expression profile associated with response to neoadjuvant chemoradiotherapy in locally advanced rectal cancer patients included miR-190b [40]. However none of these studies explored mechanism of action of miR-190b and its targets did not have been well described contrary to miR-190 that could interfere with VEGF-mediated angiogenesis [41]. Morevover *miR-190b* has not been selected by previous microarray breast cancer studies [16, 17, 19, 42] so we tried to decipher its properties in breast cancer. By treating MCF7 and T47D cell lines with estradiol, we demonstrated that *miR-190b* is not directly regulated by this hormone whereas it seems particularly deregulated in ER^+^ breast tumors. We could speculate that expression of *miR-190b* is controlled by other mechanisms like ER-signaling pathways independent of estrogen but it remains to be demonstrated [43, 44]. Transfection experiments with anti-miR-190b in MCF7 and T47D cell lines, or with mimic of *miR-190b* in MDA-MB-231 cell line have shown that *miR-190b* has probably no effect on cell proliferation. Nevertheless, if the mechanisms of its expression regulation like its exact role in oncogenesis of ER^+^ breast cancers are elucidated, *miR-190b* could become a very interesting biomarker in ER^+^ breast cancers as it has the advantage to be highly expressed in this subtype and therefore easy to detect by RT-qPCR. We could speculate that *miR-190b* would be used as a circulating biomarker for minimal residual disease follow-up in hormone-dependent breast cancers to detect therapeutic resistance and early relapses.

Last but not least, a high expression of *miR-190b* was associated with a prolonged MFS and EFS in breast tumors, independently to ER status. To date *miR-190b* just appears in one study using global microRNA expression profiling to identify markers of recurrence in ER^+^ patients receiving tamoxifen [45]. Ten highly significant miRNAs including *miR-190b* could discriminate the patient samples according to outcome but this result was not confirmed in two validation cohorts. More interesting, *miR-190b* and its function have been explored in a very recent study in human hepatocellular carcinoma [46]. The authors have showed that up-regulation of *miR-190b* could play a role for decreased IGF-1 that induce insulin resistance in hepatocellular carcinoma. IGF-1 appears to be a direct target of *miR-190b* and another study have demonstrated that IGF-1 and ER expressions are raised in breast cancer cases which were likely to develop tamoxifen resistance [47]. Taking into account our present work and these two recent studies, we argued that the link between *miR-190b* and tamoxifen resistance could be very interesting to study in breast cancers.

## Conclusion

This study identified *miR-190b* as the highest up-regulated miRNA in ER^+^ breast cancers compared to ER^−^ tumors and to normal breast tissues. Surprisingly, expression of *miR-190b* is not directly regulated by estradiol. Using synthetic miRNA to mimic or to antagonize *miR-190b*, we demonstrated that *miR-190b* does not affect the proliferation of transfected breast cancer cell lines. However *miR-190b* affects MFS of breast cancer patients. Even if *miR-190b* exact role in breast carcinogenesis and regulation expression mechanisms remain to elucidate, this microRNA seems to be specifically expressed in ER^+^ breast cancers at higher level that all others miRNAs and could therefore represent a new biomarker of interest for the follow-up of this subtype of tumors.

## Additional files

Additional file 1: Table S1. Relative mRNA expression level of 804 miRNAs studied in normal, ER+ and ER- breast tissues. For each miRNA, we give the number of samples analyzed (Nb), the median and the range (min and max) of mRNA level relative to normal breast tissue samples, the median of cycle threshold (Ct) obtained by RT-qPCR, the fold change (FC) between ER+ and ER- tumors and the *p*-value associated to Mann-Whitney's test (ns for not significant when *p*-value >0,05). The miRNAs are alphabetically ranked.
Additional file 2: Table S2. Relative mRNA expression level of 333 informative miRNAs in normal, ER+ and ER- breast tissues. For each miRNA, we give the number of samples analyzed (Nb), the median and the range (min and max) of mRNA level relative to normal breast tissue samples, the median of cycle threshold (Ct) obtained by RT-qPCR, the fold change (FC) between ER+ and ER- tumors and the *p*-value associated to Mann-Whitney's test (ns for not significant when *p*-value > 0,05). All miRNAs indicated with an asterisk following their name, all miRNAs with a median of values of cycle threshold (Ct) obtained by RT-qPCR upper to 30 in the three groups and all miRNAS with a Ct upper to 30 in at least 60 % of samples were filtrered. The miRNAs are alphabetically ranked.
Additional file 3: Figure S1. Expression profiles of 12 miRNAs significantly up-regulated in ER^+^ compared to ER^−^ breast tumors. Five expression profiles were identified: *miR-26a1* expression representative of *let-7b*, *miR-101-1*, *miR-30c2*, *miR-143*, *miR-26b*, *miR-376c* and *let-7a1* in **A**, *miR-30e* expression in **B**, *miR-193b* expression in **C**, *miR-342-5p* expression in **D** and *miR-190b* expression in **E**. For each time, the mRNA levels were normalized such that the median value of normal cells was of 1 (mean ± SEM, n = 3). Only the *p* values analyzing the differences in miRNA expression between ER^+^ and normal breast tissue and between ER^−^ and normal breast tissue by the Mann-Whitney’s test are given.
Additional file 4: Figure S2. Expression profiles of 8 miRNAs significantly down-regulated in ER^+^ compared to ER^−^ breast tumors. Five expression profiles were identified: *miR-654-3p* expression representative of *miR-18b* and *miR-18a* expression in **A**, *miR-146a* expression representative of *miR-203* in **B**, *miR-92a1* expression in **C**, *miR-155* expression in **D** and *miR-1244* expression in **E**. For each time, the mRNA levels were normalized such that the median value of normal cells was of 1 (mean ± SEM, n = 3). Only the *p* values obtained by the Mann-Whitney’s test analyzing the differences in miRNA expression between ER+ and normal breast tissue and between ER- and normal breast tissue are given.
Additional file 5: Table S3. Relative miRNA expression levels of the 30 selected miRNAs in breast cancer. Results in breast tumors are expressed as the median (range) of miRNA level relative to normal breast tissues. For each miRNA, we report the fold-change (FC) between breast tumors and normal breast tissue and the *p*-value associated to Mann–Whitney test (ns for not significant). These 30 miRNAs are ranked according their expression level in ER+ compared to ER- breast tumors (Table 3).
Additional file 6: Figure S3. Effects of estradiol on expression levels of *miR-190b* and *pS2* in MCF-7 (A) and T-47D (B). Cell lines were treated with estradiol (E2) or vehicle during the indicated time and mRNA levels were measured by RQ-PCR normalized to *RNU44* (mean ± SEM, n = 3). For each time, the mRNA levels were normalized such that the median value of control cells was of one (horizontal line).
Additional file 7: Figure S4. *MiR-190b* does not interfere with proliferation in MCF7, T47D and MDA-Mb-231 cell lines. MCF7 (A) and T-47D (B) cell lines were transfected with antagomir against *miR-190b* whereas MDA-MB-231 cell line (C) was transfected with miR-190b mimic. Cytotoxicity was evaluated by MTT colorimetric test at indicated times (mean ± SEM, n = 3).

## Abbreviations

Ct: Cycle threshold
EFS: Event free survival
ER: Estrogen receptor
FC: Fold change
MFS: Metastasis free survival
RT-qPCR: Reverse transcriptase quantitative polymerase chain reaction
RT-PCR: Reverse transcriptase polymerase chain reaction
