# Supplementary figures and images for: MiR-190b, the highest up-regulated miRNA in ERα-positive compared to ERα-negative breast tumors, a new biomarker in breast cancers?
Source: BMC Cancer. 2015 Jul 5;15:499. doi: 10.1186/s12885-015-1505-5 (PMC4491222; doi:10.1186/s12885-015-1505-5)

## Slide 1
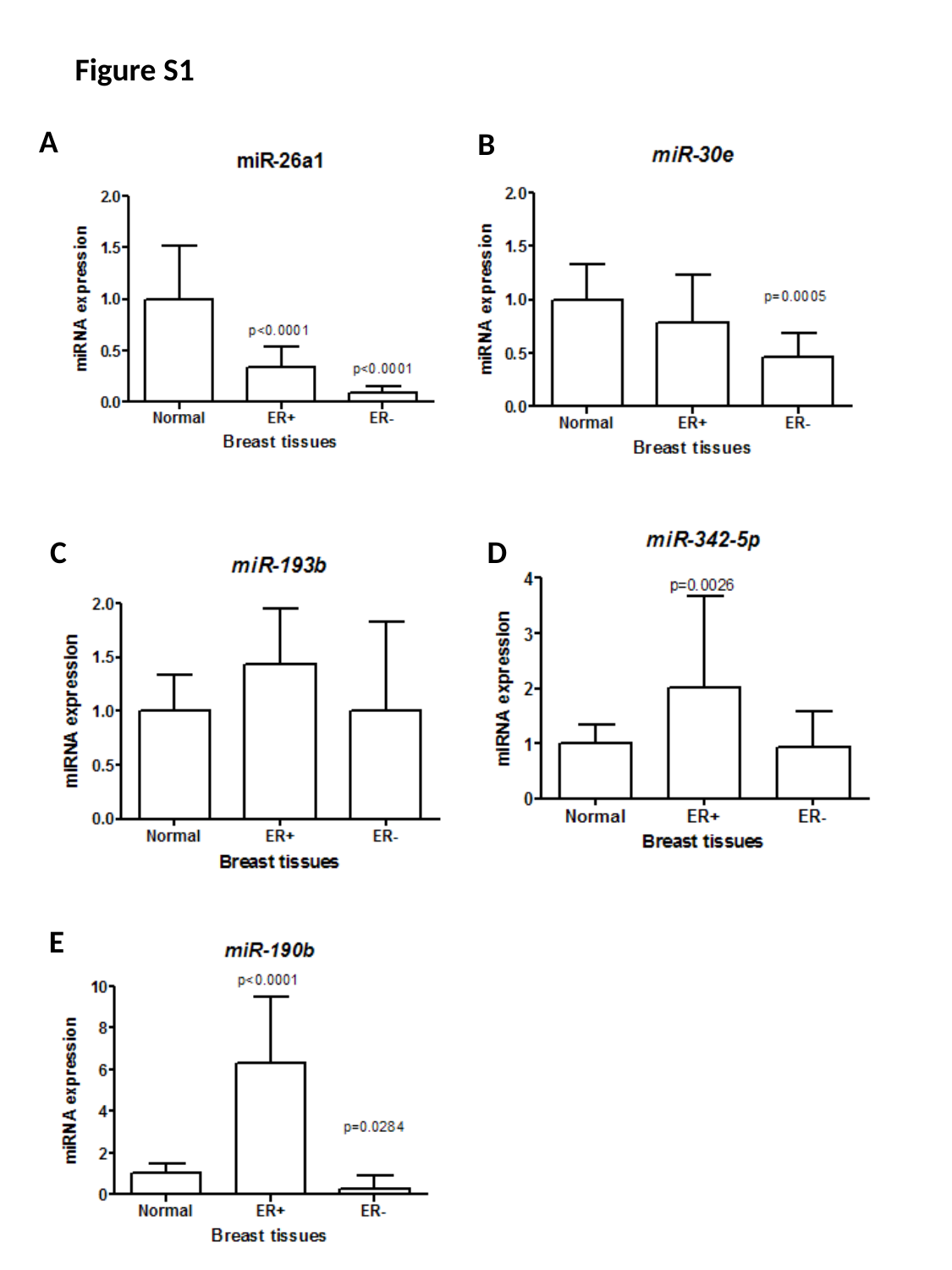

Figure S1
A
B
C
D
E

Supplement: Additional file 3: Figure S1. — Expression profiles of 12 miRNAs significantly up-regulated in ER+ compared to ER− breast tumors. Five expression profiles were identified: miR-26a1 expression representative of let-7b, miR-101-1, miR-30c2, miR-143, miR-26b, miR-376c and let-7a1 in A, miR-30e expression in B, miR-193b expression in C, miR-342-5p expression in D and miR-190b expression in E. For each time, the mRNA levels were normalized such that the median value of normal cells was of 1 (mean ± SEM, n = 3). Only the p values analyzing the differences in miRNA expression between ER+ and normal breast tissue and between ER− and normal breast tissue by the Mann-Whitney’s test are given. [file 12885_2015_1505_MOESM3_ESM.ppt]

## Slide 1
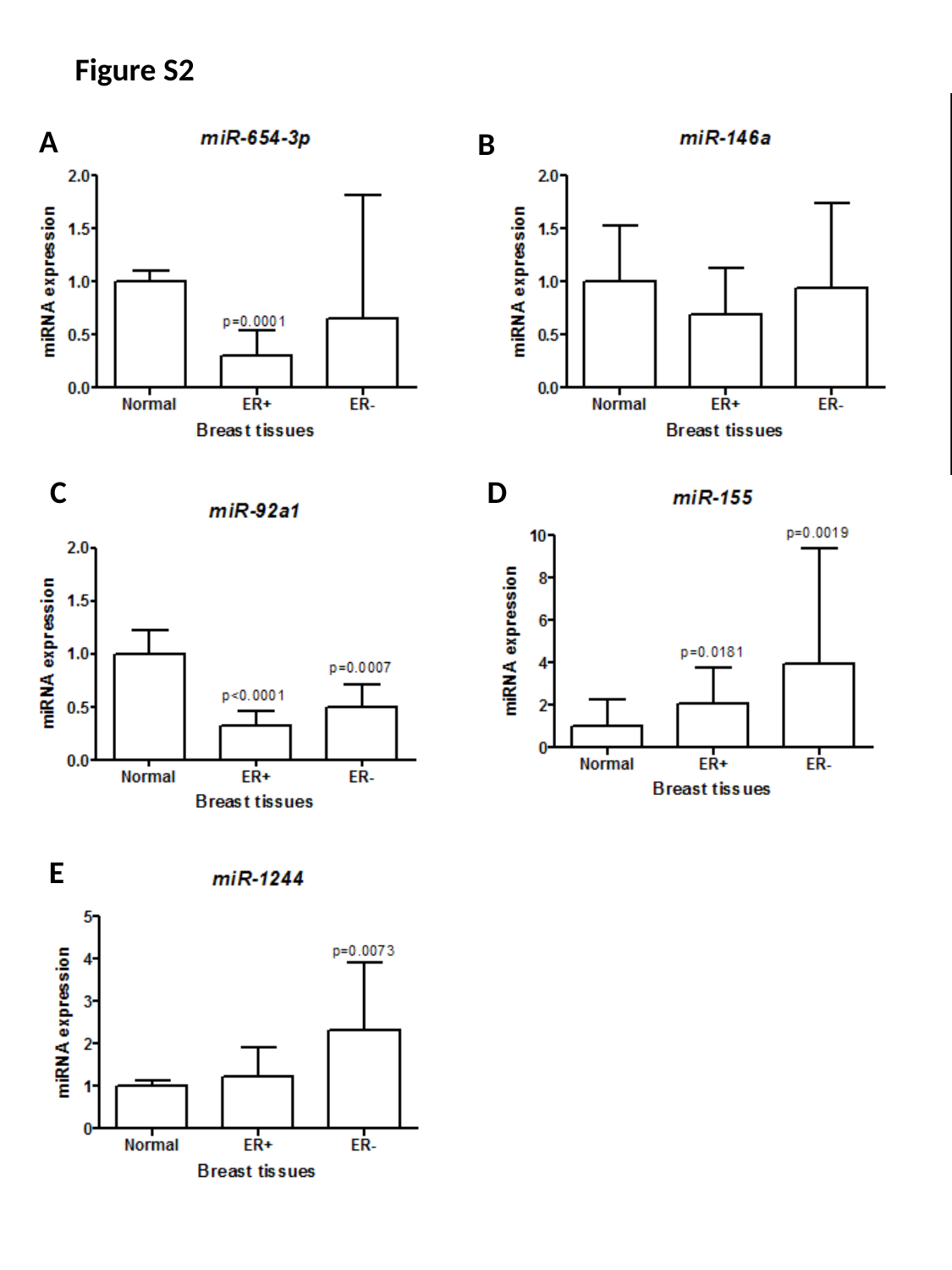

Figure S2
A
B
C
D
E

Supplement: Additional file 4: Figure S2. — Expression profiles of 8 miRNAs significantly down-regulated in ER+ compared to ER− breast tumors. Five expression profiles were identified: miR-654-3p expression representative of miR-18b and miR-18a expression in A, miR-146a expression representative of miR-203 in B, miR-92a1 expression in C, miR-155 expression in D and miR-1244 expression in E. For each time, the mRNA levels were normalized such that the median value of normal cells was of 1 (mean ± SEM, n = 3). Only the p values obtained by the Mann-Whitney’s test analyzing the differences in miRNA expression between ER+ and normal breast tissue and between ER- and normal breast tissue are given. [file 12885_2015_1505_MOESM4_ESM.ppt]

## Slide 1
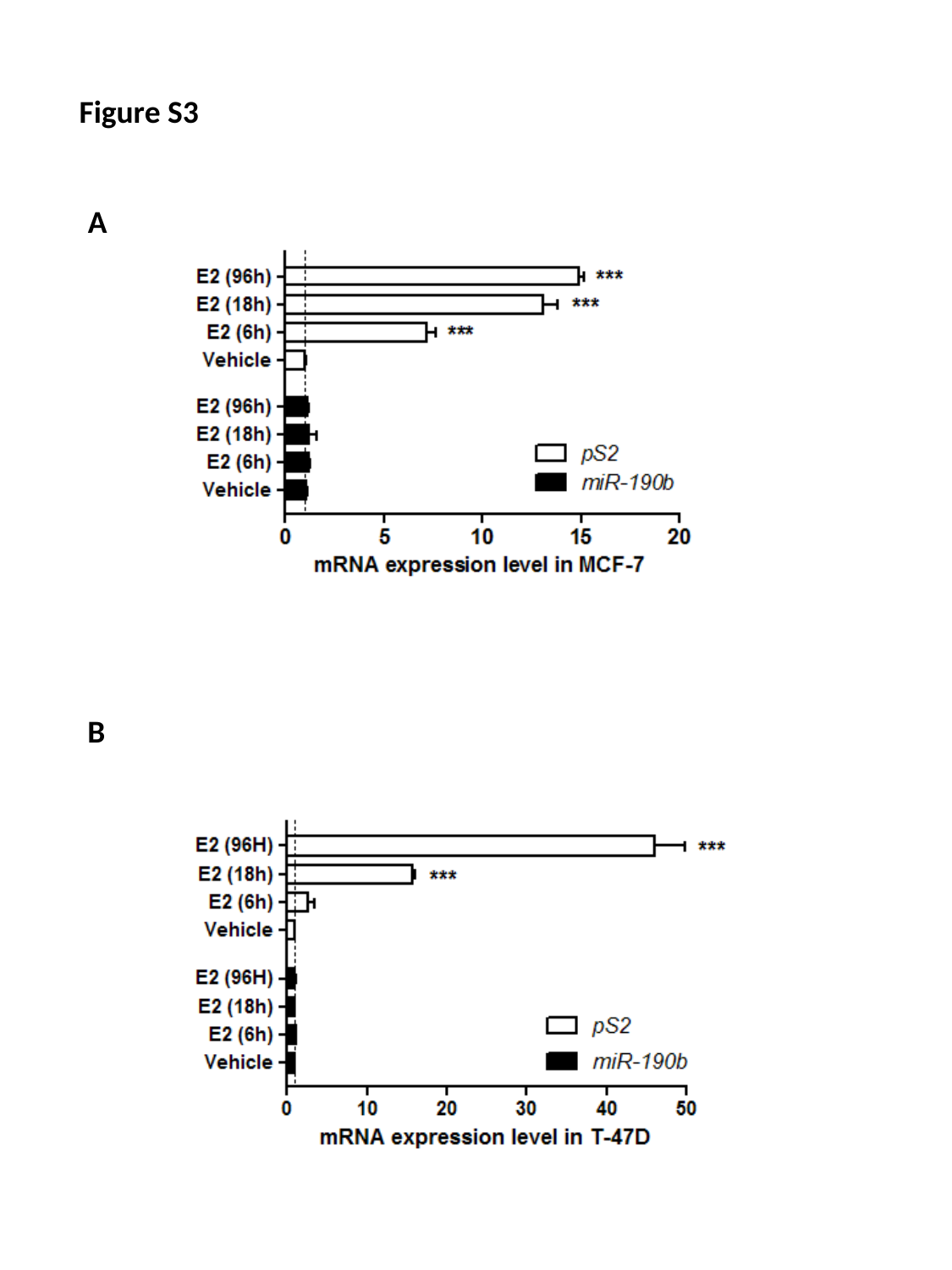

Figure S3
A
B

Supplement: Additional file 6: Figure S3. — Effects of estradiol on expression levels of miR-190b and pS2 in MCF-7 (A) and T-47D (B). Cell lines were treated with estradiol (E2) or vehicle during the indicated time and mRNA levels were measured by RQ-PCR normalized to RNU44 (mean ± SEM, n = 3). For each time, the mRNA levels were normalized such that the median value of control cells was of one (horizontal line). [file 12885_2015_1505_MOESM6_ESM.ppt]

## Slide 1
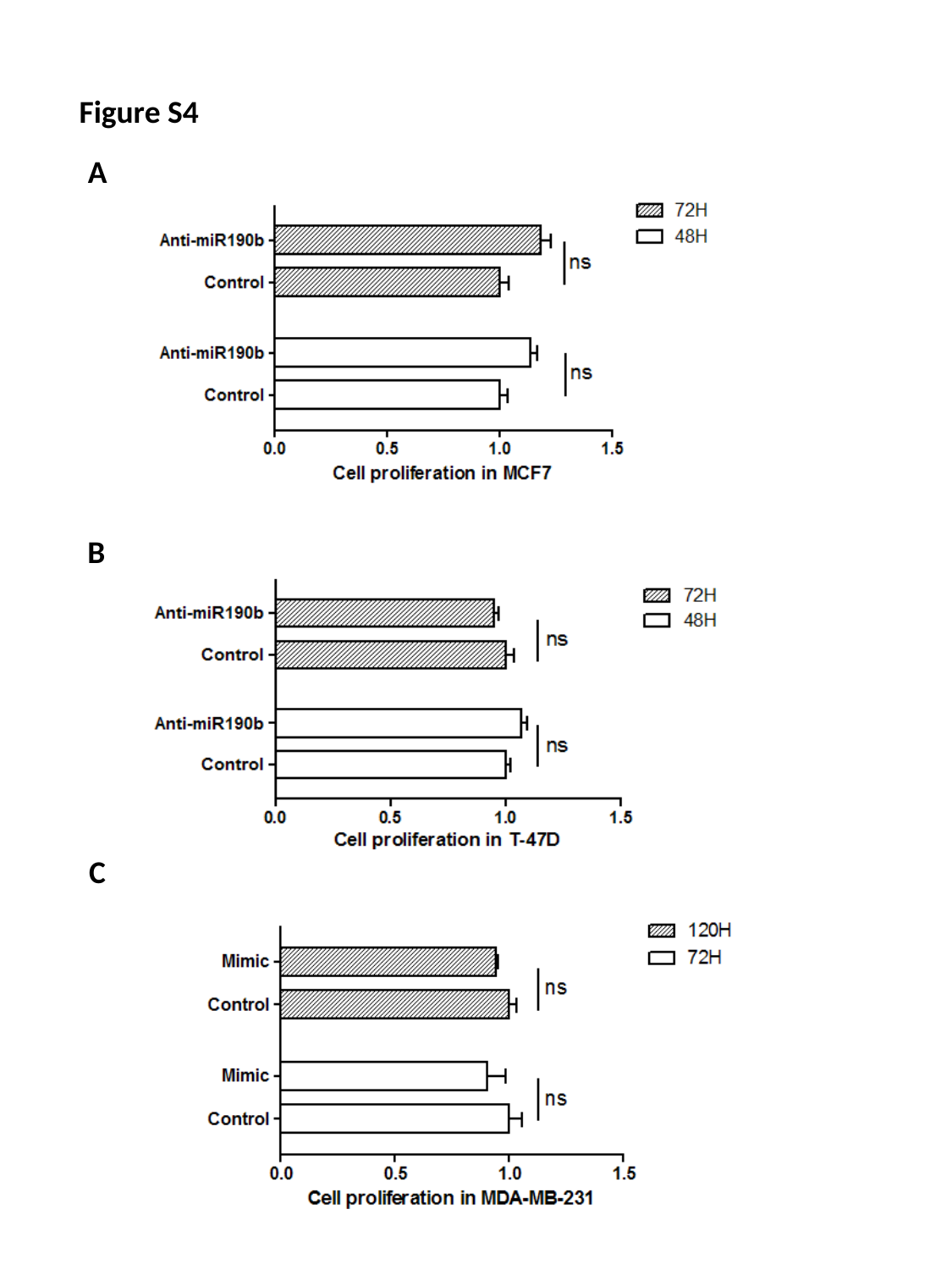

Figure S4
A
B
C

Supplement: Additional file 7: Figure S4. — MiR-190b does not interfere with proliferation in MCF7, T47D and MDA-Mb-231 cell lines. MCF7 (A) and T-47D (B) cell lines were transfected with antagomir against miR-190b whereas MDA-MB-231 cell line (C) was transfected with miR-190b mimic. Cytotoxicity was evaluated by MTT colorimetric test at indicated times (mean ± SEM, n = 3). [file 12885_2015_1505_MOESM7_ESM.ppt]
